# Supplementary material for: Mechanisms governing the pioneering and redistribution capabilities of the non-classical pioneer PU.1
Source: Nat Commun. 2020 Jan 21;11:402. doi: 10.1038/s41467-019-13960-2 (PMC6972792; doi:10.1038/s41467-019-13960-2)
Supplement: Supplementary file 7 — Source data [file 41467_2019_13960_MOESM7_ESM.zip › Source_Data/FigureS2/FigureS2B_MotifScanOutput/KmeansCluster8/homerResults/motif3.similar.html]

motif3

## Information for motif3

C
T
G
A
A
T
G
C
G
C
A
T
A
C
G
T
A
G
T
C
G
A
C
T
T
A
C
G
G
A
T
C
G
A
C
T
C
G
A
T
G
A
C
T
A
G
C
T
G
A
C
T
  
Reverse Opposite:  

C
T
G
A
T
C
G
A
C
T
G
A
C
G
T
A
C
T
G
A
C
T
A
G
A
T
G
C
C
G
T
A
A
C
T
G
C
G
T
A
C
G
T
A
T
A
C
G
G
A
C
T
  

|  |  |
| --- | --- |
| p-value: | 1e-90 |
| log p-value: | -2.083e+02 |
| Information Content per bp: | 1.715 |
| Number of Target Sequences with motif | 179.0 |
| Percentage of Target Sequences with motif | 11.84% |
| Number of Background Sequences with motif | 683.4 |
| Percentage of Background Sequences with motif | 1.51% |
| Average Position of motif in Targets | 101.7 +/- 43.9bp |
| Average Position of motif in Background | 98.9 +/- 76.6bp |
| Strand Bias (log2 ratio + to - strand density) | -0.1 |
| Multiplicity (# of sites on avg that occur together) | 1.07 |
| Motif File: | file (matrix) reverse opposite |

### Similar de novo motifs found

|  |  |  |  |  |  |  |  |
| --- | --- | --- | --- | --- | --- | --- | --- |
| Rank | Match Score | Redundant Motif | P-value | log P-value | % of Targets | % of Background | Motif file |
| 1 | 0.849 | C G T A A T G C A C G T A C G T A G T C A C G T A T C G A G T C C G A T | 1e-63 | -145.672576 | 16.27% | 4.49% | motif file (matrix) |
| 2 | 0.804 | C T G A C T A G A G T C C T G A A C T G C T G A G C T A A T C G G C A T C T A G | 1e-46 | -107.136282 | 14.62% | 4.69% | motif file (matrix) |
| 3 | 0.819 | C G A T C G A T C G T A A C T G A T G C C G T A A C T G C G T A C G T A A C T G A G C T | 1e-43 | -99.753133 | 4.76% | 0.45% | motif file (matrix) |
| 4 | 0.880 | C G T A C G T A A C T G A G T C C G T A A C T G C G T A C G T A | 1e-42 | -98.055215 | 8.80% | 1.95% | motif file (matrix) |
| 5 | 0.812 | C T A G G T A C C G T A A C T G C G T A C G T A A C T G A G C T | 1e-38 | -89.595316 | 15.67% | 5.95% | motif file (matrix) |
| 6 | 0.790 | A C T G A G T C C T G A A C T G C G T A C G T A A T C G | 1e-36 | -83.310530 | 22.62% | 10.96% | motif file (matrix) |
| 7 | 0.806 | A T G C A C G T A C G T G T A C A C G T A T G C C G A T C G A T A C G T A C G T | 1e-20 | -46.466673 | 4.56% | 1.11% | motif file (matrix) |
| 8 | 0.805 | A G T C C G A T A C G T A G T C A G T C A C T G A G T C G A C T C G A T A C G T A G T C A G C T | 1e-16 | -38.474731 | 1.46% | 0.09% | motif file (matrix) |
